# Prevalence of High-Risk HPV Subtypes and Efficacy of the HPV Vaccine in Preventing Cervical Epithelial Lesions: Survey and Insights from a German Study

**DOI:** 10.3390/life13081637

**Published:** 2023-07-27

**Authors:** Mahmoud Abbas, Jan de Jonge, Olaf Bettendorf

**Affiliations:** 1 Department of Pathology, Gerhard-Domagk Institute for Pathology, University Muenster, Domagkstrasse 17, 48149 Muenster, Germany; 2 Department of Pathology, Institute for Pathology and Cytology (IPN), 48465 Schuettorf, Germany; j.dejonge@pathologie-zytologie.de (J.d.J.); o.bettendorf@pathologie-zytologie.de (O.B.)

**Keywords:** HPV vaccine, precancerous, cervical cancer

## Abstract

Background: Historically, cervical cytology has been the standard method for detecting dysplastic cervical changes. However, extensive research has established that human papillomavirus (HPV) infection is a primary cause of these changes, necessitating a shift in screening and preventive strategies towards the molecular detection of high-risk HPV subtypes. To combat HPV infection, prophylactic vaccines have been developed, including the nonavalent, quadrivalent, and bivalent vaccines. An essential criterion for an effective HPV vaccine is to provide comprehensive coverage against the most prevalent high-risk HPV types associated with cervical cancer, ensuring optimal efficacy in preventing cervical lesions. Long-term protection against these types is crucial for effective prevention strategies; Material and Methods: A cohort of 210,510 women’s samples was included in the analysis conducted within one year of implementing a screening program in Germany. The screening program involved the molecular detection of high-risk HPV subtypes, targeting specific age groups. The cohort comprised 63,710 women below 35 years of age and 146,800 women aged 35 years and above. The selection of high-risk HPV subtypes followed the guidelines provided by Becton-Dickinson. This study focused exclusively on cases with a documented history of vaccination, which were categorized into two main groups: Group I consisted of vaccinated individuals under 35 years old (12,765 cases), while Group II comprised vaccinated individuals aged 35 years and above (296 cases); Results: The HPV types HPV56/59/66 were found to be widely distributed across all age groups, with certain age groups exhibiting a higher incidence compared to HPV16 and HPV18. Similarly, HPV35/39/69, along with HPV31 and HPV45, were also observed to have a broad distribution among women. The incidence of high-grade squamous intraepithelial lesions (HSIL), including both CIN2 and CIN3, varied between 0.076% and 0.5% across all age groups, regardless of the individuals’ vaccination status; Aim of the study: Our study provides valuable insights into the distribution, incidence, and prevalence of various high-risk HPV subtypes, including HPV56/59/66, HPV33/58, HPV35/39/68, and HPV45, in relation to precancerous cervical lesions. These subtypes are not adequately covered by the currently available HPV vaccines. Addressing the discrepancies between the prevalent HPV subtypes and existing vaccines is crucial in developing an ideal HPV vaccine that offers comprehensive protection. Tailoring screening programs and vaccination strategies to the local distribution of HPV subtypes is essential for effective prevention. By raising awareness and implementing targeted preventive measures, including vaccination, we can significantly reduce the incidence of precancerous and cancerous cervical lesions.

## 1. Introduction

Cervical cytology, introduced by Dr. Papanicolaou in the 1920s to 1940s, has long been the standard method for detecting cervical dysplastic changes. However, despite its use, HPV infection remains the primary cause of cervical lesions in sexually active women, prompting the need for alternative screening and prevention strategies. Various screening approaches have been proposed and implemented, such as the recommended use of cervical cytology alone every three years or HPV subtyping of high-risk groups every five years in the USA, while European guidelines discourage cervical cytology as a co-testing method [1]. The prevalence and incidence of high-risk HPV subtypes in women over 26 years old have been extensively documented [2,3]. Prophylactic vaccines, including nonavalent, quadrivalent, and bivalent vaccines, have been developed to target HPV infection [4]. However, effectively addressing the wide range of high-risk HPV subtypes associated with tumor development remains a challenge. Several studies have proposed specific screening recommendations for HPV-vaccinated women, with regional and vaccination-based variations. In Europe, there is a bivalent vaccine (Cervarix) and a nonavalent vaccine (Gardasil 9). HPV infection with high-risk genotypes is associated with other dysplastic mucosal changes in the ano-genital and oro-pharyngeal regions [5]. The bivalent HPV vaccine was produced to protect against HPV16- and HPV18-associated cervical lesions. The quadrivalent HPV vaccine was produced to protect against low-risk subtypes HPV6- and HPV 11-associated, and the high-risk subtypes HPV16- and HPV18-associated cervical lesions. The nonavalent HPV vaccine was produced to prevent persistent infection and precancerous lesions caused by common low-risk (HPV6 and HPV11) and high-risk HPV subtypes like 16, 18, 31, 33, 45, 52, and 58 [5]. The ideal HPV vaccine should cover at least the majority of those linked to tumor development, the so-called high-risk subtypes, and it should protect long-term. [6]. Many current studies have tried to perform specific recommendations for a first line of prevention with screening of HPV-vaccinated women, as tried by Italian screening [7], which included primary HPV screening starting at the age of 30 years old with a screening interval of 5 years; but, they have recommended the universal application of these recommendations only when herd immunity is reached. The publications from Asia recommended other screening methods and other methods of vaccination. We believe that the spreading of HPV subtypes is different from one nation to another and, consequently, there is the differential appearance of cervical dysplastic lesions. This study aims to investigate the distribution of HPV high-risk subtypes among women in Germany, with a particular focus on identifying the most prevalent subtypes associated with precancerous and cancerous cervical lesions. By elucidating the HPV subtypes with the greatest risk and evaluating their inclusion in commonly used vaccines, this research contributes to the development of an effective HPV vaccine capable of providing substantial protection against cervical lesions. By employing HPV vaccination and screening approaches, such as cytology and/or HPV testing, precancerous lesions can be identified and treated prior to the development of cervical cancer. To the best of our knowledge, this study represents the first comprehensive effort of its kind in the literature. Its findings have the potential to significantly enhance the efficacy of HPV vaccination and reduce the incidence of precancerous and cancerous cervical lesions.

## 2. Material and Methods

A total of 210,510 samples were collected from women over the course of one year following the implementation of a novel screening system for the detection of cervical dysplastic changes. The screening protocol encompassed different approaches depending on the age group: conventional cervical cytology for women under 30 years old, a combination of conventional cytology and molecular detection of high-risk HPV subtypes for suspicious cases between 30–34 years old, and co-testing (combining conventional cytology with molecular detection of high-risk HPV subtypes) for women aged 35 and above. The study included 63,710 cases under 35 years old and 146,800 cases at or above 35 years old. Both conventional cytological techniques and molecular detection with subtyping of high-risk human papillomavirus (HPV-HR) were performed using the Becton-Dickinson role-based method (117,765 samples) and the PapilloCheck HPV test (5579 samples). A subset of cases underwent immunocytochemical examination using CINtecPlus and L1-Capsid. Excluded from the analysis were approximately 84,000 samples from individuals under 35 years old who lacked a justifiable medical indication for HPV subtype detection (Group I) or were tested elsewhere. This study focused specifically on the high-risk HPV subtypes designated by BD. While genotypes 16 and 18 historically account for 70% of invasive cancer cases worldwide, their prevalence is decreasing due to increasing vaccination rates. Genotypes 31, 33, and 58 have a similar risk of CIN3+ as genotype 18, whereas genotypes 51, 35, 39, 68, 56, 59, and 66 have a substantially lower risk. Vaccination history was taken into account, and the study population was divided into two main groups: Group I comprised individuals vaccinated under 35 years old (12,765 cases), while Group II consisted of individuals vaccinated at or older than 35 years old (296 cases). The study aimed to assess the incidence and distribution of high-risk HPV subtypes in these groups and correlate the findings with histopathology results following colposcopy, to identify subtypes associated with precancerous or cancerous lesions not covered by commonly used vaccines. Ethical approval for the study was obtained from the Ethics Committee of the Medical Association in Hannover, Germany. Informed consent was waived due to the anonymized and retrospective analysis of patient records, ensuring compliance with data protection protocols.

## 3. Results

 *I*  *Distribution of HPV high-risk subtypes within the cohort samples (Table 1):*

Under the age of 35 years old, there is a higher incidence of HPV 56/59/66 (0.037%) compared to HPV 16 and HPV 18.

Between the ages of 35–40, there is a higher incidence of HPV 16 (1.23%) than other HPV high-risk subtypes, including HPV 56/59/66, HPV 35/39/69, and HPV 31.

Among individuals aged 41–50, there is a higher incidence of both HPV 56/59/66 and HPV 16 compared to the other studied subtypes.

For those between the ages of 51–60, there is a higher incidence of HPV 56/59/66 than other studied HPV high-risk subtypes, including HPV 16 and HPV 35/39/69.

Individuals older than 60 years old exhibit a higher incidence of HPV 16 compared to other studied HPV high-risk subtypes.

 *II*  *The incidence of HPV 18 within the cohort samples (Table 1):*

In women older than 35 years old, the incidence is 0.25% (373 cases). Common associations are observed with other HPV high-risk subtypes, including 16 (29 cases), 45 (13 cases), 31 (17 cases), 52 (19 cases), 59 (1 case), 51 (12 cases), 33/58 (14 cases), 56/59/66 (6 cases), and 35/39/68 (2 cases).

 *III*  *Relation between histopathological diagnosis, age of women, and vaccination status within the cohort (Table 2):*

Under the age of 35, the incidence of CIN3 is 0.31%, higher than other preinvasive lesions (CIN1 and CIN2).

Between the ages of 35–40, the incidence of CIN3 is 0.5%, higher than other preinvasive lesions (CIN1 and CIN2).

Between the ages of 41–50, the incidence of CIN1 is 0.36%, higher than other preinvasive lesions (CIN2 and CIN3).

Between the ages of 51–60, the incidence of CIN1 is 0.25%, higher than other preinvasive lesions (CIN2 and CIN3).

In women older than 60, the incidence of CIN1 is 0.098%, higher than other preinvasive lesions (CIN2 and CIN3)

 *IV*  *Relation between cytological group and associated HPV high-risk subtypes in vaccinated women aged 35 and above (Table 3):*

In cases categorized as normal or negative for intraepithelial lesion or malignancy, there were incidences of HPV 35/39/68 (3 cases) and HPV 52 (2 cases).

Among cases with a low-grade squamous intraepithelial lesion (LSIL), there were incidences of HPV 31, 56/59/66, and 35/39/68, each with one case.

Cases with a high-grade squamous intraepithelial lesion (HSIL) showed association with HPV 31, 51, 16, and 56/59/66, each with one case.

There was one case of a high-grade glandular intraepithelial lesion (AIS) associated with HPV 16.

 *V*  *Relation between cytological group and association with HPV high-risk subtypes in vaccinated women under 35 years old (Table 4):*

Among cases categorized as normal or negative for intraepithelial lesions or malignancy, there was association with HPV 56/59/66, HPV 33/58, and HPV 35/39/58.

Cases with a low-grade squamous intraepithelial lesion (LSIL) exhibited association with HPV 18, 51, 52, 35/39/68, 56/59/66, and 33/58.

Cases with a high-grade squamous intraepithelial lesion (HSIL) showed association with HPV 52.

## 4. Analysis of the Results

There are noteworthy findings, which include the identification of high-risk HPV subtypes (56/59/66) in two cases with histopathological diagnosis of CIN1 and one case with CIN3. Additionally, a case with HPV-HR (35/39/58) was detected in association with a histopathological diagnosis of CIN1.

Other significant findings in the results section encompass:

The distribution of high-risk HPV (HPV-HR) within the study population, revealing the prevalence of distinct HPV subtypes across various age groups.

The incidence of cervical lesions (CIN2, CIN3) in relation to age, providing insights into the occurrence of precancerous cervical abnormalities among different age cohorts.

Observations in vaccinated women aged 35 years and older, encompassing the identification of specific HPV subtypes and their correlation with histopathological diagnoses of a cervical intraepithelial neoplasia (CIN).

Findings in vaccinated women under the age of 35, highlighting the presence of HPV-HR subtypes and their association with cytological abnormalities, although without histopathological examination in some instances.

Overall, these findings contribute to the enhanced comprehension of HPV infection prevalence and its impact, as well as the efficacy of vaccination in reducing the incidence of HPV-related cervical lesions.

## 5. Discussion

Cervical cancer prevention has witnessed a shift towards the utilization of HPV testing as the primary screening method, surpassing cervical cytology (Pap. Test), due to its heightened sensitivity and potential ability to prevent a greater number of cervical cancer cases. The molecular detection of HPV high-risk subtypes has prompted changes in cervical cancer screening practices. It is crucial to adapt the prevention strategies for cervical dysplastic changes associated with these subtypes accordingly. Vaccination emerges as the optimal approach and should be regarded as the primary preventive measure for controlling infectious diseases. Widely implementing vaccination programs can prove highly effective and economically feasible compared to the treatment and management of cervix carcinoma.

Human papillomavirus (HPV) belongs to a group of more than 200 double-stranded DNA viruses, with approximately 40 mucosal types commonly transmitted through sexual activity [8]. HPV infection is responsible for approximately 5% of all human cancers, contributing to over 600,000 new cases worldwide each year [9]. Recognizing the significance of HPV vaccination, the Advisory Committee on Immunization Practices recommended the use of a 9-valent human papillomavirus (HPV) vaccine (9vHPV), among the three available HPV vaccines for routine vaccination during its February 2015 meeting [10]. These vaccines consist of DNA-free virus-like particles that are non-infectious and non-oncogenic, providing long-lasting immunity.

Australia took a pioneering step in 2007 by implementing a school-based HPV vaccination program targeting girls aged 12 to 13 years, becoming the first country to do so. The Advisory Committee on Immunization Practices (ACIP) also recommends HPV vaccination for females aged 13 through 26 years and males aged 13 through 21 years who were not previously vaccinated [11]. The 9vHPV vaccine, known as Gardasil 9, includes virus-like particles (VLPs) of HPV 6, 11, 16, and 18, along with additional VLPs of HPV 31, 33, 45, 52, and 58. On 10 December 2014, the Food and Drug Administration (FDA) approved 9vHPV for females aged 9 through 26 years and males aged 9 through 15 years [12].

However, despite international recommendations for vaccine coverage, our study revealed low vaccination rates. Only 20% of women under 35 years old (12,765 cases) received the HPV-HR vaccine, and a mere 0.2% (296 cases) of women at or above the age of 35 were vaccinated. The utilization and coverage of HPV vaccination remain inadequate, indicating a lack of established vaccination programs in schools and in low-income countries. For instance, in the USA, in 2012 [13], only 33.4% of adolescent females and 6.8% of adolescent males aged 13–16 years received the recommended three doses of the HPV vaccine.

In our study, we identified a correlation between precancerous cervical lesions and HPV-HR subtypes such as 56/59/66, either individually or in combination with HPV-HR 51, HPV33/58, or HPV45. While HPV 16 is commonly associated with HPV-associated lesions, our findings contrast with previous research by Markowitz LE et al., in 2014 [11]. Similarly, Saraiya M. in 2015 [14] reported that HPV 16 or 18 accounts for 66% of cervical cancers, while five additional types contribute to approximately 15% of cases. Furthermore, Hariri S. and colleagues [15] highlighted that approximately 50% of CIN2+ lesions are caused by HPV 16 or 18, with HPV 31, 33, 45, 52, or 58 responsible for 25% of these lesions. The two HPV-HR genotypes, HPV 16 and HPV 18, account for 70% of cervical cancer cases worldwide, while HPV 31, 33, 45, 52, and 58 contribute to another 18% of cases [16]. Surprisingly, our study detected cases of HPV-16 infection without associated dysplasia.

Additionally, we observed an incidence of HPV18 in women under 35 years old, with a rate of 0.006% (four cases, one of which received vaccination). Among women above 35 years old, the incidence was 0.25%, and all cases were non-vaccinated individuals. Although indirect immune cross-reactivity has been observed, providing partial protection against HPV31 with 4vHPV and HPV31, 33, and 45 with 2vHPV vaccines, the strength and duration of the antibody responses evoked by these vaccines are lower than those targeting specific vaccine genotypes. This observation challenges the clinical efficacy, as suggested by Luciano M. and colleagues in 2016 [17]. A comprehensive population-based study conducted in Sweden examined the incidence of invasive cervical cancer (ICC) categorized by HPV type, encompassing 2850 confirmed ICC cases spanning the years 2002 to 2011. The study revealed two age groups exhibiting higher incidence rates: one within the 30–45 age range and another among older age groups, approximately 70–80 years [18]. A Norwegian study aligns with these findings, as HPV 16, 18, and 45 emerged as the predominant HPV types detected in cervical cancer cases, predominantly among women aged 35–40. Conversely, other oncogenic HPV types, including 31, 33, 35, 39, 52, 58, and 73, displayed a higher prevalence among older age groups [19]. Notably, two cross-sectional studies investigating HPV distribution in European ICC cases also reported a significant association between young age and HPV 16-, 18-, and 45-positive cervical cancer cases [20], bolstering our own findings where the mean age for these three types ranged approximately from 44 to 45 years. These findings underscore the importance of incorporating HPV 45 into primary prevention strategies targeting HPV-related cervical diseases.

According to a Swedish study, a significant majority (85.3%) of screen-detected cervical cancers were found to be associated with HPV types 16, 18, 31, 33, 45, or 52. The inclusion of the remaining eight HPV types covered by most screening tests only marginally increased the prevalence by 1.5% [21]. Similarly, a Norwegian study reported that the eight additional types were detected in only 1.4% of screen-detected cancers, while the majority (93.0% or 66 out of 71 cases) were caused by HPV types 16, 18, 31, 33, and 45 [19]. In Europe, it has been reported that the majority of cervical cancer cases are caused by five high-risk HPV types, namely HPV 16, 18, 31, 33, and 45 [22].

A recent population-based HPV prevalence study conducted in the Nordic region by Nygård et al., suggested that HPV screening tests, in the post-vaccination era, may perform better when restricted to the HPV types covered by the nonavalent vaccine. Screening for all 14 HPV types could potentially result in a suboptimal balance of harms and benefits [23].

Considering these findings, it is imperative to develop targeted HPV vaccination strategies against the newly identified high-risk subtypes.

## 6. Conclusions

Our study sheds light on the prevalence, incidence, and distribution of HPV high-risk subtypes responsible for precancerous lesions, including HPV56/59/66, HPV33/58, HPV35/39/68, and HPV45, in a substantial number of cases. Notably, this study stands alone in the current literature in its comprehensive exploration of these HPV subtypes in relation to the occurrence and incidence of precancerous cervical lesions. The findings underscore the disparities between the prevailing HPV vaccines and the actual prevalence of HPV infections among women. This serves as a crucial initial stride towards the development of an ideal HPV vaccine capable of targeting all significant subtypes. We stress the importance of implementing tailored screening programs as the primary preventive measure, accompanied by vaccination initiatives employing appropriate vaccines that address locally prevalent HPV infections as a complementary secondary defense strategy in each nation. By addressing these concerns, we aim to enhance the uptake of HPV vaccination among healthcare providers and the general population, consequently reducing the frequency of precancerous lesions and their progression to cervical cancer.

## 7. Limitation of This Study

The HPV DNA test BD Onclarity combines the results for HPV56/59/66, HPV33/58, and HPV35/39/68, which poses limitations in genotype analysis for CIN3 and determining relevant HPV types for inclusion in an HPV vaccine. Notably, HPV types 56, 59, and 66 are not prevalent in cervical cancer cases. Furthermore, HPV type 33 exhibits a higher prevalence compared to HPV 58, while HPV type 35 is more prevalent in cervical cancer than types 39 and 68. These findings highlight the importance of accurately identifying and targeting high-risk HPV genotypes to enhance the effectiveness of HPV vaccines. This will be followed in our institute by undertaking further studies and follow-up of these cases.

## Figures and Tables

**Table 1:** Distribution of HPV high-risk subtypes within the cohort samples.

HPV/Age Group	<35 Y	35–40 Y	41–50 Y	51–60 Y	>60 Y
56/59/66	0.037%	0.93%	1.1%	0.99%	0.66%
16	0.03%	1.23%	1%	0.7%	0.67%
35/39/69	0.017%	0.76%	0.87%	0.58%	0.42%
51	0.017%	0.36%	0.33%	0.25%	0.16%
31	0.01%	0.76%	0.66%	0.42%	0.28%
52	0.007%	0.397%	0.4%	0.3%	0.2%

**Table 2:** Relation between histopathological diagnosis, age of women, and vaccination status within the cohort.

Histopathological Diagnosis/Age Group	<35 Y	35–40 Y	41–50 Y	51–60 Y	Over 60 Y
Cervical intraepithelial neoplasia (CIN I)	0.13%	0.35%	0.36%	0.25%	0.098%
CIN II	0.23%	0.42%	0.26%	0.22%	0.078%
CIN III	0.31%	0.5%	0.33%	0.13%	0.076%
Squamous cell carcinoma (Sq.c.ca)	0.005%	0	0.009%	0.021%	0.023%
Adenocarcinoma in situ (AIS)	0.002%	0.005%	0.01%	0.005%	0.002%
Endocervical adenocarcinoma	0.002%	0.005%	0.014%	0.007%	0.004%
Endometrium carcinoma	0	0	0.003%	0.034%	0.07%
Without dysplasia	0.19%	0.5%	0.56%	0.51%	0.26%

**Table 3:** Relation between cytological group and associated HPV high-risk subtypes in vaccinated women aged 35 and above.

HPV-Status/Cyto-Group	I and IIa (274)	IIp (1)	IIID1 (9)	IIID2 (7)	IVa-p (1)	IVa-g (2)
Negative	142	0	2	2	0	1
16	1	0	0	1 (without Dysplasia)	0	1 (CIN III)
45	1	0	0	0	0	0
51	1	0	0	0	0	0
52	1	0	0	0	0	0
35/39/68	3	0	1	0	0	0
56/59/66	1	0	1	0	1 (CIN II)	0
31	1	0	0	0	0	0
31 + 56/59/66	0	0	1 (CIN II)	0	0	0
31 + 51	0	0	0	1 (CIN II)	0	0
Unknown	123	1	4	3	0	0

**Table 4:** Relation between cytological group and association with HPV high-risk subtypes in vaccinated women under 35 years old.

HPV-HR Status/Cyto-Group	Group I and IIa (12,156)	Group IIp (102)	IIID1 (353)	IIID2 (75)
Negative	17	100	3	1
56/59/66	4	1	1 (CIN I)	0
33/58	1	0	0	0
35/39/58	3	0	0	0
51	0	0	0	1
58	0	0	0	1
31	0	0	0	0
51 und 33/58	0	1	0	0
52 + 56/59/66	1	0	0	0
45 + 56/59/66	1	0	0	0
52 + 51 + 35/39/68	0	0	1	0
18	0	0	1	0
52 + 51 + 35/39/68 + 56/59/66	0	0	1 (CIN I)	0
51 + 56/59/66	0	0	1 (CIN III)	0
56/59/66 + 31 + 35/39/68	0	0	1 (CIN I)	0
52 + 45	0	0	0	1 (CIN III)
Unknown	12,129	0	344	71

## Data Availability

The dataset used and/or analyzed during the current study are available from the corresponding author on reasonable request.
